# Smashing, Shaming, or Polite Fun and Joy? How Workplace Humor Influences Positive Well-Being in South Korean Workplaces

**DOI:** 10.3389/fpsyg.2021.682183

**Published:** 2021-08-04

**Authors:** Hee Sun Kim, Barbara Plester

**Affiliations:** ^1^Department of Business Administration, College of Humanities & Social Sciences Convergence, Yonsei University, Seoul, South Korea; ^2^Business School, University of Auckland, Auckland, New Zealand

**Keywords:** humor, psychological well-being, hierarchy, honorifics, Korea

## Abstract

Humor is contextual, ambiguous, and varies within cultures but is widely associated with positive outcomes such as well-being and happiness. While humor is universal and enhances interpersonal relationships which can benefit psychological well-being, we argue that humor can also be diminish psychological well-being in Confucian-based, South Korean workplaces. Our research questions asks: *how do hierarchical workplace relationships influence shared humor and positive well-being in Korean workplace contexts?* Our contextual, ethnographic research includes in-depth field observations and semi structured interviews in three Korean organizations. Traditional Confucian-based cultures value face-saving, trust, and harmony while emphasizing formality and hierarchy. Korean honorifics maintain harmony, hierarchy, and politeness which creates benefits for group processes and influences the sharing of humor. Humor is enacted in accordance with workers' hierarchical status which has a significant impact upon the types of humor shared and the responses available to subordinate employees. Investigating these dimensions in Korean workplaces we argue that honorifics and hierarchy influence humor interactions in complex ways that have implications for psychological well-being.

## Introduction

Humor is a contextual phenomenon that exists in most cultures (Berger, 1987). Intercultural competence is globally important, ambiguous, and nuanced (Deardorff, 2015). Humor is similarly ambiguous, nuanced and inconsistent which means that it may vary in forms, performances, and motives. Often perceived as a positive interaction, humor may help people to develop interpersonal relationships (Cooper, 2008), and shared laughter may help to establish similarity and familiarity with others (Brown and Levinson, 1978). Humor is seen to promote psychological well-being (PWB) as it can be a coping mechanism to reduce stress and provide relief from everyday tensions (Freud, 1960) and is beneficial in defusing emotional events (Ridanpää, 2019). However, humor is complex and may be interpreted differently by the actors involved. It is also culturally specific and contextual, therefore humor may only be perceived as *funny* when it is deemed appropriate for the context. Although humor is generally considered beneficial to PWB, it may also be “tendentious” (Freud, 1960), provocative, cause emotional harm, and even damage workplace relationships (Plester, 2016; Kim and Plester, 2019). Therefore we argue that it is important to research and analyze humor within its cultural context to understand the situational nuances and dynamics that cause it to flourish or fail with varying impacts on PWB.

In our contextual study, we focus upon the Confucian-based culture that underpins South Korean organizations and is based on complex language structures that reflect status differences between communicators (Brown, 2011). While honorifics and specific titles may be used to help Koreans to communicate more respectfully in accordance with their relational status (McBrian, 1978), hierarchical structures are complex and communication protocols can be ambiguous within organizations. Our paper examines how the Confucian based values of hierarchy, formality, and polite respect may influence humor interactions in Korean organizations and impact upon PWB. We focus on hierarchical organizational relationships as we enquire: *how do hierarchical workplace relationships influence shared humor and positive well-being in Korean workplace contexts?* Our contribution is in emphasizing cultural meanings and significances (Hatch, 2012) of organizational humor that have significant implications for PWB in modern Korean workplaces.

## Psychological Well-Being

The *absence* of psychological disorders (such as anxiety and depression) and the *presence* of positive attributes creates PWB (Ryff, 1989; Ryff and Keyes, 1995). Eudaimonic well-being extends the definition of well-being “beyond feeling good to include functioning well” (der Kinderen et al., 2020, p.1). PWB is particularly salient to workplace functioning and performance and is linked to success and health (Robertson and Cooper, 2011). The basic structure of psychological well-being is centered around the “distinction between positive and negative affect and life satisfaction” (Ryff, 1989, p. 1,070). Ryff established six long term dimensions of PWB, these being: self-acceptance, positive relations with others, autonomy, environmental mastery, purpose in life; and personal growth and these reflect enduring life challenges. Ryff (1989) does warn however, that demographic differences such as culture ethnicity, history, and class may lead to different and even competing conceptions of well-being.

While many factors of PWB are established and understood, the relationship between humor and PWB humor is nascent but studies are emerging showing that humor has a positive relationship with PWB as it can generate benefits in self-efficacy, self-concept, positive affect, optimism while simultaneously reducing stress, depression, and anxiety (Martin et al., 1993; Crawford and Caltabiano, 2011). Such effects are not firmly established in humor studies linked with PWB. It is the complexity and contextual nature of humor coupled with a variety of humor styles [see Martin et al. (2003) and Ruch and Heintz (2018)] and personalities that can produce ambiguous results and prompts calls for further research examining humor's influence upon PWB.

## Humor and PWB

The multifaceted nature of humor includes cognitive, social, emotional, and behavioral aspects (McCreaddie and Payne, 2014). Psychological literature dating back to Freud (1960) has established that humor is a coping mechanism that can relieve stress, and Freud's early work underpins a key group of humor theories that focus on humor as a beneficial relief and release from everyday tensions. Freud argued that laughter is liberating both physically and psychologically. Building on Freud's work, psychologists have identified that good humor creates positive effects that strengthen self-esteem while reducing levels of depression and anxiety (Lefcourt and Martin, 1986; Martin, 2006). Psychologist Martin (2006) argues that increased levels of humor enhance positive self-concept and self-esteem and that this also occurs when people experience extreme negative life events. The benefits of alleviating anxiety, stress reduction, and mitigating existential threat can all improve PWB (Martin et al., 1993). Therefore, humor can be a beneficial coping mechanism to deal with highly emotional interactions, which assists PWB especially in times of crisis (Ridanpää, 2019). Sustaining a humorous perspective about life's vagaries and crises helps to create stability that improves resilience and PWB (Plester, 2009; Cann and Collette, 2014). The relationship between managers and employees influences humor use and PWB. In a study of 2,498 Australian employees, Wijewardena et al. (2017) found that high quality manager- employee relationships prompted more positive humor use and supported healthy emotional regulation at work.

However, while these positive influences on PWB make for popular findings and are commonly assumed, there are also a variety of maladaptive elements of humor that can be self-defeating and hostile (Plester, 2016). Hostile humor tends to focus on other people, it may be aimed or targeted and therefore does not enhance well-being (Kuiper et al., 2004). Humor can disparage, belittle, debase, demean, humiliate, and victimize others (Zillmann, 1983; Billig, 2005) and such unpleasant aspects of humor can have detrimental effects on those who may be the target of this type of joking. Freud (1960) also acknowledged that “tendentious” (controversial) humor can allow an expression of an idea or viewpoint that might be normally “unsayable.” While this might offer an amusing release to the joker, victims of this type of humor are likely to suffer negative emotions and reactions. Therefore effects on PWB may differ between different actors in humor instances, depending on their role or position in the humor exchange.

Bitterly et al. (2017) argue that humor is risky because unsuccessful humor can harm a persons' status at work whereas successful humor can imply both confidence and competence which may increase a joke teller's status. They note the important role of humor in shaping hierarchy and interpersonal relations within groups. Gender also plays a role in workplace humor. Gender stereotypes influence workplace humor in that males who use humor at work are awarded higher status than those who do not use humor. Conversely, females who use humor at work are ascribed lower status than females who do not use humor (Evans et al., 2019).

Workplace research has established that banter (Plester and Sayers, 2007), clowning and practical jokes [see Plester and Orams (2008)] and the dark side of humor (Plester, 2016) involve complexity and ambiguity and evoke a range of responses and feelings. Investigating both positive and dark features of humor illuminates some specific contextual factors that strongly influence humor and PWB for workers and managers. Contextual factors may include the physical environment in which humor is enacted and/or the characteristics of those involved in the humor. Therefore context is an important factor in determining whether or not humor is well-received and works toward increasing PWB; whether humor may have the opposite effect and degrade PWB, or whether it may actually achieve both of these at the same time. A key contextual dimension to humor, and highly pertinent to this current study, is the cultural context for humor. The cultural context incorporates norms, values and relational factors that guide and influence humor participants (Marra and Holmes, 2007) and effects their PWB.

## Cultural Context and Workplace Humor

Workplace humor can be a positive experience (Ruch, 2008) that creates a sense of commonality (Alden et al., 1993) and happiness among people (La Fave et al., 1976). However, humor use and interpretation is highly contextual and based on the underlying cultural values of the society and the interacting work group (Marra and Holmes, 2007). Humor is a multifaceted term which includes the mental process, creation, and emotional responses of, and toward funny or laughable gestures and words (Martin and Ford, 2018). This can vary between different cultures and Tanaka (2018) illustrates that shared laughter in Japanese contexts may signal interactional issues that need to be resolved rather than amusement.

Humor may help to increase innovation and creativity (Isen et al., 1987), assist interpersonal communication processes within the workplace (Holmes, 2000), achieve resilience (Cheung and Yue, 2012), and describe individual differences (Ruch et al., 2018). Therefore, organizations often encourage employees to use humor but may not consider that there may be potentially negative impacts (Morreall, 1983). Workplace humor may exclude some people (Plester and Sayers, 2007), or can be interpreted negatively creating conflict and workplace disruption (Plester, 2016).

Both organizational culture (Plester, 2016) and the society in which the organization is based may influence humor significantly (Davis, 2016). This is especially significant in globalized workplace contexts where people from different cultures interact and work together. Cultural differences have a strong impact on humor perception and those from Eastern cultures do not favor humor or use it as a coping strategy compared with workers from Western contexts (Jiang et al., 2019). Although inquiry into national culture may generate a stereotype of a culture, it can also provide a useful initial framework to explore and refine a particular culture's characteristics (Gale and Vance, 2012) that may explain everyday workplace practices and behaviors.

Cultures influenced by Confucian philosophy may approach the idea of humor differently to those from Western contexts (Yue et al., 2016). Humor in Confucian cultures may be perceived as a threat to the authority of organizational power-holders and therefore be seen as inappropriate (Kim and Plester, 2019). People exchanging humor interact from their normative orientation and background which influences the humorous relationship and the management of “face” is particularly important in Korean contexts (Kim, 2018). Harmony is also psychologically important in collectivist cultures (Yamagishi et al., 1998). Therefore, workplace humor studies must be situated and examined through an appropriate cultural lens that appreciates how cultural behaviors are shaped by national traditions (Azevedo, 2020) and we now turn to Confucianism's influence in Korean workplaces, significant to our current study.

## Confucianism in Korean Workplaces

Korea is one of the East Asian countries that is considered to be strongly influenced by Confucian practices and values, affecting the daily behaviors and PWB of Korean people (Deuchler, 1992; Choi, 2010). Confucianism is a philosophy of ethics that emphasizes the importance of interdependency and harmony within the society, derived from the ancient Chinese scholar Confucius (Deuchler, 1992; Yao, 2000). It is perceived that *hierarchy* maintains beneficial societal harmony and individuals are expected to adopt relational roles based on age and family-based relationship structures that prescribes unequal power between individuals (Yao, 2000; Lun and Bond, 2006). Harmonious relationships are important in Korean society and workplaces and have also been positively linked with improved PWB particularly for older people (Chiang et al., 2013).

While Confucianism is traditionally a Chinese philosophy, other countries such as Korea, Vietnam, and Japan are also influenced by Confucian values (Duncan, 2002). This is also supported by Hofstede's (1984) study, where Korea shares similar cultural characteristics with other East Asian cultures that embed Confucian-based values. However, Confucian values and how these values are reinforced through rituals, rites, and communication, may differ across Asian cultures, therefore cultural ideas or concept may be interpreted differently in the variety of Confucian contexts (McSweeney, 2002).

Confucian values are embedded in Korean society and reflect its traditional authoritarian rule (Rowley and Bae, 2003) including expectations of respect, hierarchy in interpersonal relationships, and collective attitudes that prioritize group goals and harmony. Interdependence and harmony is encouraged, where “superiors” are expected to perform a duty of care (to subordinates) and subordinates must display respect and obedience in familial, societal and workplace relationships (Yao, 2000) in order to prioritize the harmony expected in most Korean contexts.

Societal and relational communication structures transfer to organizational contexts (Rowley and Bae, 2003). In Korea, communication processes between employees of different hierarchical status at work are also significantly influenced by societal Confucian values. Subordinate employees are restricted in their communication modes and silence is particularly preferred as it signals obedience (Lim, 1999). A system of honorifics that denote relational social positions is commonly used in society and at work and this is considered a form of respect that reinforces the status of higher-ranked people (McBrian, 1978; Hwang, 1991).

## Confucian Politeness, Hierarchy, and Humor

Much of the extant literature on the relationship between culture and humor has a westernized focus. Western organizational research (Holmes, 2000, 2006; Holmes and Marra, 2002; Plester and Orams, 2008) has emphasized the reduction of hierarchical levels when humor is shared at work. Cooper (2008) suggests that humor influences the quality of interpersonal relationships between people regardless of their hierarchical differences. While humor still occurs in Confucian contexts, including Korea (Kim and Lee, 2009; Jung, 2014), hierarchical dynamics in humor interactions are not fully investigated in Confucian contexts (Jung, 2014). The Western tradition of using workplace humor that reduces hierarchical differences may be less relevant to Confucian-based cultures with hierarchical structures that prioritize collective identity and harmony.

Within Confucian societies, age is revered and influences relational interactions (Yao, 2000) creating a family-like, often patriarchal relationship where people must behave in accordance with their prescribed hierarchical status (Deuchler, 1992). This means Korean workplace relationships intersect with Confucian societal expectations guiding behavior and formal or informal interactions. Relational hierarchy is important to maintain harmonious organizational life in Korean workplaces and hierarchy also influences communicative norms (Shim et al., 2008). Language is governed by specific titles of address and the appropriate form of address for a superior or a subordinate is highly dependent on their relational status. For example, the word “nim” (direct translation, “sir” or “madam”) may be the most appropriate title to use toward an organizational superior, rather than the less formal mode of address—“ssi” that translates to “Miss” or “Mr” in English.

In line with formal protocols for addressing colleagues at work, the use of humor is carefully considered in Korean organizational communication. The indirectness of humor creates ambiguity which is a feature of humor (Wood et al., 2011). It seems that the indirectness of humor can help to maintain some forms of politeness (Brown and Levinson, 1978) which is important in Korean society where politeness is interlinked with using honorifics (House and Kasper, 1981; Upadhyay, 2003). With relational Confucian notions of hierarchy and politeness at the fore, we explore workplace humor and its impact upon PWB within a specific cultural context. We reflect cohesive cultural identifications while simultaneously exposing problematic humor aspects within an evolving modern (Korean) culture increasingly impacted by Western workplace influences. Our study analyses humor and its effect upon workplace relationships and PWB in Korean organizations underpinned by Confucian cultural traditions. With this objective we investigate: *how do hierarchical workplace relationships influence shared humor and positive well-being in Korean workplace contexts?*

## Methodology

### Multi-Voiced Interpretivist Approach

In order to explore the complex relationship between organizational humor and PWB associated with Confucian values in Korean workplaces, we adopt a qualitative, interpretive approach (Creswell, 2003). Such an approach allows us to understand meaningful social action in context (Denzin and Lincoln, 2005) and permits multi-voiced, multiple participant realties to generate rich, nuanced interpretations (Alvesson and Sköldberg, 2000). As organizational humor may be subjective and interpreted differently by everyone (Holmes, 2006) our multi-voiced interpretivist approach (Alvesson, 2010; Cunliffe et al., 2014) helps us to investigate diverse contextual interpretations of humor and to present different voices for analysis. This helps to understand the complex relationships within studied organizations by providing rich stories and descriptions of the studied phenomena (Bryman and Bell, 2015). In keeping with our interpretivist methodology, we make no claims of generalizability of our findings.

### Data Collection Methods

We collected data from three different Korean companies using participant observation and semi-structured interviews to represent the interpersonal and subjective nature of organizational humor. The empirical observations and interviews took place over 4 months (March to June 2014). We selected three different companies to extend our learning (Stake, 2013) and our participant companies vary in work characteristics and size. These companies were recruited through the researcher's personal networks, where the researcher was introduced to managers of potential participant companies (thus initially not directly connected or personally related to the participant companies). This method of recruitment is an important part of gaining research access within Korean organizational context, as interpersonal relationship (based on collectivistic ideals) is valued. Each company's Chief Executive Officer was provided with a detailed information sheet and consent form prior to the research, to explain about the research process and assure confidentiality. These documents were reviewed and authorized by the Human Ethics Committee of the researcher's institution. Furthermore, individual participants were also provided with information sheet and consent form prior to the research. The three companies operate in Information Technology (IT), online gaming, and manufacturing industries. We assigned the pseudonyms *Truscene, Mintrack*, and *Wisepath*, to preserve confidentiality and also gave interview participants pseudonyms. Summative tables are provided below, detailing participant companies (Table 1) and individual interview participant's demographic details (Table 2).

**Table 1:** Participant companies.

**Company**	**Industry**	**Size**	**Number of levels of hierarchy**	**Age range**
Truscene	Information technology	49	7	20–40's
Mintrack	Online gaming	33	8	20–60's
Wisepath	Manufacturing	63	10	20–60's

**Table 2:** Interview participants.

**Company**	**Truscene**	**Mintrack**	**Wisepath**	**Total**
**Gender**
Female	5	4	2	11
Male	20	10	5	35
**Age group**
20's	12	7	0	19
30's	9	6	1	16
40's	4	1	1	6
50's	0	0	3	3
60's	0	0	2	2
**Organizational position**
Sawon (entry-level)	10	6	4	20
Juim (Manager)	5	1	0	6
Daeri (Deputy section chief)	3	2	0	5
Gwajang (Section chief)	3	3	0	6
Chajang (Deputy department manager)	2	1	0	3
Bujang (Department manager)	1	1	0	2
Isa (Deputy managing director)	0	0	2	2
Jeonmu (Managing director)	0	0	1	1
Bu-sajang (Vice-CEO)	0	0	0	0
Sajang (CEO)	1	0	0	1
**Tenure**
0–2	17	9	3	29
3–4	0	3	0	3
5–6	3	0	1	4
7+	5	2	3	10

One researcher fully immersed in each company for a period of 1 month in order to contextualize employees' experiences and perceptions. Observation was conducted 5 days per week, and sometimes during weekends if the participants invited the researcher to join extra company and/or social activities. Participant observation focused on language and humor interactions between organizational members and affective behaviors and responses. Detailed field notes were recorded throughout the research. The researcher engaged in organizational activities during working and non-working hours, and undertook work tasks such as assisting in writing company reports, translating documents (from Korean to English), serving beverages at company events, and engaging in factory work in order to assimilate to company life. The researcher also participated in afterhours drinking with participants, and weekend social gatherings to observe participant behavior diverse situations. To further understand the subjective experience and implications for employees, both formal and informal (impromptu) interviews were used. Our field researcher is ethnically Korean and Korean language was used in all interviews, being the most comfortable language for our participants (Welch and Piekkari, 2006). Forty-six semi-structured interviews were conducted, and all participation was voluntary. Approximately 1–2 h were used to conduct each interview, and hand-written notes and voice recorder was used to collect interview data. After the research period, participants were provided with a summary of their data, and were given the opportunity to provide further information through member check method.

### Analysis

Thematic analysis was used to understand patterns and themes within the data providing flexibility and appropriate for the exploratory nature of this project (Braun and Clarke, 2006). Our analysis concentrates on individual and group humor interactions, reactions, responses, and emotive aspects that may contribute to PWB. In particular, Confucian values, ideals, perceptions and their impact on employees in humor instances are investigated in detail and we include employee interpretations of their significance and meaning to their well-being. Relevant synonyms and key words in transcripts and observation data were analyzed carefully (Auerbach and Silverstein, 2003). Observations and interview excerpts were coded into categories and further categorized into themes.

Analysis comprised multiple phases, from initial data collection and first categorisations to an all-encompassing review of combined data. Data were stored and organized using NVivo 12 Plus. Our four different stages of analysis include:

Stage 1: Initial analysis- during data collection period
Stage 2: After data collection- analysis within each company
Stage 3: Coding and codes from all companies combined
Stage 4: Combined analysis arranged and recoded into themes

## Findings

### Contextual Information

Context is important for humor and workplace relationships, therefore findings detailing the three companies are presented first. Confucian-based values of hierarchy, politeness and respect were apparent in all of our observation data and we analyzed language, honorifics, gestures, and non-verbal actions. Participants from all companies engaged in humor in their everyday interactions. Interview data extended our observations through capturing participants' perceptions of their organizational humor and its influence upon their workplace relationships and PWB.

Understanding of Confucian notions of hierarchy and levels of formality emerged through analyzing our observational data of interpersonal interactions and language used by participants. Korean language conveys different degrees of formality and relative hierarchical status is signaled through honorifics and personal titles (similar to other cultures that utilize diverse linguistic forms depending on the speaker's relational status). Korean workers assess their relative (hierarchical) position to others in an interaction and consider this context before engaging in communicative exchanges (McBrian, 1978). Organizational position and age of other people are important factors in determining the relational status or position of the communicators. Our participants used different titles for each other based on organizational position as well as age-based societal titles such as “*ssi*” (“mr.” or “miss”), “*nim*” (“sir” or “madam”) followed by the addressee's last name and first name. Managers (or those with “superior” status) from all three companies used more casual language (than subordinates) and had more freedom in addressing subordinates. Examining the different cultural practices and the language used by the participants helps to identify relational hierarchy which emphasizes the underlying Confucian-based value of maintaining harmonious relationships necessary for PWB.

### Organization One: Truscene

Truscene is an Information Technology (IT) company with 49 employees. Only eight employees are female and workers' ages range from 20 to 40's, with most of the senior management aged over 40. Truscene has a seniority system that promotes workers based on organizational tenure, which is common in Korean organizations. Of the three companies, Truscene displayed the most complex organizational culture. The cultural behaviors and cues that we observed, differed from the managerially endorsed and espoused organizational culture [see Schein (1985) and Schein (2004)]. Furthermore perceptions of organizational culture from senior managers contradicted clarifications from junior employees. Most of the senior managers claim that Truscene has a horizontal, non-patriarchal culture but this is not confirmed by subordinates. Observational data that included verbatim recording of interactions, showed that subordinate employees use strict honorifics and titles adhering to the formal structure that includes the managers' full name and organizational position using the formal Korean address- “nim.” In contrast, managers mostly addressed subordinates informally using the subordinates' first name- “ssi” or their last name and organizational position. We interpret this as a power signal demarking the hierarchical differences between workers- where managers get to be casual in their address while subordinates must use formal structures denoting hierarchical “superiority.” This junior worker explains the need to use formal language:

*In Truscene, it's different to the overseas (Western) cultures. We need to address seniors formally, like their organizational positions should be used, full organizational positions, and they care a lot about age and hierarchy. So I think it's quite strict in that way*. (Carnelian, 24, Truscene).

Carnelian identifies the difference between Truscene and Western organizations. She also explains that seniors “*care a lot about age and hierarchy*” signifying the importance of adhering to strict, formal communication toward her superiors and suggesting that workers' age is a key factor. Elders are highly revered and respected in Korean culture and honoring traditional values important to seniors is an important factor in fostering collective workplace harmony and well-being.

### Organization Two: Mintrack

Mintrack is a small online gaming company with 33 employees and only four female (administrative) staff. On average, workers are aged 20–30, while senior managers range from 40 to 60 years. Mintrack has a looser organizational system with a high degree of individual freedom. For example, organizational members are able to decide on their work hours (either a 9 or 10 am start), and freely shift their work stations, to engage in work tasks and team meetings as needed. Workers use casual language and managers use their subordinate's first name or “ssi” to address them. Subordinates use personal appellations such as “*hyung*” (“older brother”) used by younger men toward senior men. Interview participants describe this casual language and work atmosphere:

*I would feel icky if people call me that (middle manager), plus it makes me look old. So unni is enough [laughs]*. (Citrine, 33, Manager).

*We're just really casual. I call everyone hyung (older brother) and it's all good here*. (Garnet, 29, Subordinate).

Both Citrine and Garnet explain that casual language is preferable to formal organizational titles, and reflects a less-hierarchical culture and warmer relationships within this organization which is perhaps more typical in gaming companies worldwide. This fosters a less traditional organizational culture and the informality allows casual workplace interactions that include more humor that enhances PWB for the younger workers.

### Organization Three: Wisepath

Wisepath is a recycling company that handles diverse precious metal. Wisepath employs 63 workers with only seven female employees. Most workers are 40–50. The company has two divisions—factory and office. The office has nine hierarchical levels while the factory has two levels. Wisepath displayed strong Confucian values and traditional organizational culture and advertises its collective culture on the company webpage, emphasizing cooperation and collaboration. Organizational members use strict forms of language (honorifics and titles) in accordance with their relational hierarchy to reinforce subordinate or superior organizational status. This conversational extract exemplifies the formal hierarchical relationship acknowledged through titles:

*Coral (64, female): I worked for quite some time, and I'm the oldest staff here I think*.

*Morgan: Yes, she is two years older than me, and senior. She's the manager. Just a two person team but she's the manager so I call her “manager”- being respectful, careful [laughs]*. (Wisepath, May 30th: Observation notes).

Although these participants are lighthearted about the formality it is closely adhered to and important to relational dynamics in this company.

### Themes and Categories

For the purpose of guiding the observation process, initial coding schemes were developed prior to data collection (Sharpe and Koperwas, 2003) based on organizational humor and Confucianism literature. Key ideas from the literature were used to construct a schedule for semi-structured interviews. In analysis, our coding scheme included 17 categories denoting types of humor, direction of humor use, hierarchical relationships, and types of language (such as formal language or honorifics) and emotional responses. Novel observations found during the data collection period were organized by new codes and categories as they emerged (Braun and Clarke, 2006). Our coded categories generated three key themes.

- ***Relational hierarchy***was created from three categories including: 1a. *organizational hierarchy; 1b. societal hierarchy*; and *1c. status ambiguity*. This theme incorporates the influence of relational and societal hierarchy on humor use and is derived from participant perceptions of organizational humor and the ambiguity they perceive in their status when humor is used at work. This ambiguity has varying impacts on their perceptions of their personal PWB particularly the dimension concerning ‘positive relations with others' (Ryff, 1989)
- ***Language and hidden meanings***is derived from categories including: 2a *honorifics* and *2b politeness*. This second theme focuses on the incongruity and indirectness of humor in interactions and the complex layers of decoding needed in interpreting humor between organizational members. In particular, the use of honorifics and different titles used in humor may convey different (and sometimes conflicting) meanings, and may reflect different interpretations toward being “polite.” This also has varying bearings on PWB for workers as it impacts their autonomy and has positive, negative, and ambiguous impacts.
- ***Saving face***derived from categories of: *3a chemyon (face)* and *3b shame* that encapsulates how humor may protect or damage the *chemyon* (face) and that shame is a factor in some humor. Saving or damaging face has some significant impacts on PWB and can create anxiety, shame and tension. Data extracts within each theme is presented below.

### Relational Hierarchy

Our data indicates that participants approach humor interactions differently according to the relational hierarchy between workers. Observations across the three participant companies recorded 163 humor instances where managers instigated and engaged subordinates in humor, and just 40 instances where subordinate employees instigated humor to a manager.

In an interview, Jet explains the nature of humor interactions from his subordinate perspective:

*Of course, I respond actively (laugh) to the boss. But it's not really that funny… well, if the same joke is shared with people my own age or position, and comparing the same joke shared with people positioned higher than me… it's different*. (Jet, 28, Mintrack).

This extract suggests that subordinates are expected to respond “*actively*” to a superior's humor, regardless of whether the joke is funny or not. Jet also implies that it is important to consider the relational status differences (“*people positioned higher than me*”) in responding to humor interactions at work. Jet's response indicates that his responses to humor are dependent on relational aspects determined by hierarchical differences and that he will force laughter to please his boss. Therefore Jet potentially sacrifices his own PWB in ensuring that his boss saves face, potentially increasing the boss' PWB, through having his joke seem successful (invoking laughter).

Participants emphasized ambiguity in determining their relational status with others, especially when sharing humor. For example, Jet is uncertain whether it is age or hierarchical position of the superior that influences his response to his CEO's humor:

*I think it's because he is the CEO. CEOs are difficult you know, since I'm just a low-level employee. But then again, it might be the age difference… he is much, much older than me. I think it's the age*. (Jet, 28, Mintrack)

Jet's confusion about organizational position or age determining his humor response may arise from the traditional Korean seniority system where age and hierarchical status are linked in organizational status. In this company the seniority system operates in a manner whereby organizational tenure, age and position are parallel, that is, senior roles are held by those with the longest tenure—who are also older. Therefore, social interaction by employees is influenced by parallel effects that consider age, organizational position, and tenure which guide responses to everyday interactions such as joking. In other words, respect is given for age, tenure, and role and this influences subordinates' response to humor. Any response other than polite laughter might be considered disrespectful. Jet has prioritized his interpersonal work relationship which may increase his PWB (Johnson et al., 2018) but at the same time the dissonance experienced in faking laugher and humor may diminish his PWB (Sosik et al., 2017).

While Jet adjusts his humor reaction in accordance with organizational hierarchy, Larimar, a manager, emphasizes the importance of age-based (societal) hierarchy when engaging in humor with other organizational members:

*Young people these days don't like old-fashioned stuff. When I'm with young ones, I have to adjust to their interests. But when I'm with my own age group, I can joke about the concerns that we have at our age*. (Larimar, 55, Manager Mintrack).

Larimar's comment suggests difficulties in intergenerational communication and emphasizes that colleagues in the same age group have similar concerns but when he interacts with younger employees, he makes adjustments. This highlights that workers of different ages are may find it more difficult to share humor successfully. However, there are no clear divisions demarking “young” and “old” groups which makes age-related interactions ambiguous and complicates humor between those in different age demographics. According to Larimar his humor with others in his age group is successful as he can joke about “age concerns” but he perceives difficulties when sharing humor with “*young people*.”

While it is difficult for subordinate employees to differentiate between age-based and role-based effects on their humor response, managers see humor exchanges as helpful in maintaining hierarchical roles:

*I would joke around and be nice to the factory workers, people around my age. It helps me to be closer, like friends. When they slack off, I also joke to put them into shape. I am the senior manager, so I need to do what I need to do*. (Jasper, 47, Manager, Wisepath).

Jasper suggests that he uses humor to perform the role of both friend and manager in the workplace. He perceives his own humor to enhance relationship-building but at the same time he believes that he uses humor as a corrective [see Butler (2015)] designed to—“*put them into shape*.” Thus, Jasper balances dichotomous roles by using humor as he aspires to be friends and boss simultaneously. It seems that this is a useful strategy for enhancing his own PWB as he feels more comfortable managing through humor. We cannot here get a sense of his employee's responses but even if they respond with laughter, as done by Jet (above), this could be through respect for his role rather than actual friendship and similar to Jet, his subordinates may experience dissonance that impacts their PWB. The ambiguity of humor and responses to humor may help managers to both “manage” and “be friends” by mitigating the tension that occurs when there are status differences between workers. However, managing factory workers of a similar age to himself compels Jasper to treat subordinates notionally as “*friends*” while maintaining control over performance. Humor gives him a vehicle to achieve both simultaneously—at least in his own perception.

In this theme it appears that humor can mitigate hierarchical differences and display friendship but what seems more compelling in these Korean organizations is that humor serves to *emphasize* hierarchical imbalances. Humor is used as a corrective to shape behavior and humor must be respectfully accepted, acknowledged, and responded to appropriately by subordinates compelled to laugh at the bosses' jokes. Our discussion will debate the impact on PWB from these conflicting influences.

### Language and Hidden Meanings

Our participants indicated that they took care with language use in humor interactions. Interview participants stressed the need to maintain politeness within Korean society and this extended to workplaces. These participants emphasize politeness and respect, even in humor:

*When it comes to communicating with a superior, you need to be careful. In Korea, you need to respect elderly people. Be polite. Things like that are considered important. And I agree… so I do things carefully. Including humor*. (Emerald, 28, Mintrack).

*Traditionally we emphasize ourselves as people from the “Eastern nation of politeness.” Thing like… respecting your elders are so important in Korea. So, I think this would influence (humor) quite a bit*. (Citrine, 33, Mintrack).

Emerald implies that humor all communication with “superiors” need to be constructed carefully, and humor may be considered disrespectful when it is exchanged with an older person. Citrine links politeness and respect to Korean cultural norms and notes the influence of such norms on humor. Her phrase “*Eastern nation of politeness*” suggests she values Korean culture and highlights that respect and politeness toward elders are culturally significant. Therefore the positive release/relief effects that can be generated through shared humor are not available to these workers who must be constrained and careful that their humor maintains the harmony and respect required in their workplaces. Again this seems to indicate a dichotomous impact upon PWB, both enhancing it through relationally appropriate behavior that is harmonious, while reducing PWB through caution, constraint and the anxiety of possibly being impolite.

We also note differences from Western cultures. Nearly half of our interview participants felt that *politeness norms* created a boundary to humor and that the notion of *politeness* had a strong influence on everyday behavior. Park (1993) identified *rules of politeness* as a Korean societal norm and our study shows this cultural norm permeating behavior and humor at all hierarchical levels. As politeness is so strongly endorsed societally, workplace humor interactions become risky as they may contravene rules of politeness and be perceived as impolite - especially when enacted by someone in a subordinate role, creating anxiety and tension around humor reactions and experiences for subordinate workers.

While most of our participants emphasize that politeness considerations should supersede humor, the use of honorifics in humor appeared to generate varied interpretations. Linguistically, honorifics is a form of indirect language in Korean society that helps to maintain politeness (Brown, 2011; Brown et al., 2014). A few participants suggested that humor may still be considered polite but only if honorifics were fully used:

*There's a formality, or a hierarchical system that needs to be maintained here… It was my first time to drink with company people […] I called everyone unni (older sister) and obba (older brother) instead of using their organizational positions to address them […] Senior managers didn't like the way I was joking around, not using honorifics and stuff*.

(Aquamarine, 20, Truscene).

Aquamarine suggests that senior managers did not approve of her joking and it seems that the lack of “*honorifics*” was part of their disapproval. While honorific use is expected for subordinate employees, managers are permitted to joke and tease without using honorifics which demonstrates differing degrees of autonomy in using humor depending upon hierarchical status.

Although using honorifics is seen as an indicator of politeness especially when used by subordinate employees, it does not always convey politeness. The following (observed) interaction shows honorifics may in fact be used in an *impolite* way in a humorous interaction. This linguistically polite (full use of honorifics and titles) humor interaction actually delivers a very pointed barb:

*Moonstone stands up from his chair, and starts to pack up his items from his work desk. He holds onto two neatly folded shirts (uniform), turns around, and bows to other workers in the office*.

*Moonstone: Thank you all for taking care of me for the last one month. I'm sorry to leave at such short notice, but I really appreciate the time I have spent here, and I will definitely cherish all of what I have learned from here. Thank you*.

*Moonstone bows his head deeply. Onyx and Bronze stand slowly, look at Moonstone frowning*.

*Onyx: Hey Bronze, look, our sir “young and rich soon to be CEO” (of a different company) is holding onto our uniform. I hope he's returning it, because our small company needs that shirt back!*

*Bronze: (laughs)*

*Onyx: Right, sir? You're giving that back I hope?*

*Moonstone stops for a moment, and puts down the shirts on the desk*.

(Truscene, April 04: Observation notes).

Moonstone is a new employee who is leaving the company after only 1 month. Onyx, a senior manager, is not happy with Moonstone's decision, and uses humor with full honorifics to address Moonstone (“*right, sir*?”). Onyx usually uses casual language (no honorifics or formal titles) to address his subordinates. Therefore, using full honorifics toward Moonstone who is hierarchically beneath Onyx (in both organizational position and age) is incongruous and sarcastically humorous. The use of honorifics in Onyx's humor implies that Moonstone is not a colleague anymore, but an outsider. On the surface, the use of honorifics may seem to maintain a certain level of societal politeness. However, the conversation between Onyx and Bronze creates a form of double-incongruity through humor and honorifics, in order to deliver the message that Moonstone is now an outsider, and show that relationships have changed. While Onyx may have achieved some relief from his disapproval through his barbed jokes, it is likely that they have negatively impacted Moonstone- but in his subordinate role he simply bows politely.

Honorific use maintains common politeness that is important to the vertical Korean societal structure (Yoon, 2004; Brown et al., 2014). Honorifics clearly denote age differences and hierarchical differences in the workplace. Therefore, humor that does not include honorifics may be risky for these subordinate employees bound by this societal/ workplace convention. However, managers are not bound to use honorifics with those beneath them and so they may instigate a freer form of humor with their subordinates. Honorifics may be incorporated into humor and used to emphasize a humorous point, but sarcastic use of honorifics would also seem to be only a prerogative of senior managers.

### Saving Face

According to participants humor may help to redefine roles and save “face” (known as “*chemyon*”). This *chemyon* theme is strongly apparent in our observation data. Across all three participating companies, 34 observed instances of humor were recorded where humor was used to save face. However, the number of interview responses regarding this topic was much lower because participants seem to consider discussing an exposure of their “shameful” moments too painful. Thus, this theme analyses mostly observational interactions with a few interview extracts in support.

Spinel teases Ivory who is hierarchically below him based on organizational position and age:

*Spinel stands up from his desk, bending his body forward as if to see Ivory better. Other members (Diamond, Emerald, and Sapphire) are whispering to each other in a joking way, pointing their fingers at Ivory, laughing at her. Ivory covers her fringe with both her hands, while seated at her desk*.

*Ivory: My fringe was chopped by a weirdo hairdresser yesterday. I need to get it fixed up*.

*Spinel: It looks stupid. You look really stupid*.

*Diamond: Yeah you look really stupid*.

*Emerald: Sorry, you kind of look stupid*.

*Ivory makes a sad expression, making eye contact with each of the others as they comment about her hair. Her gaze stops at Spinel. Spinel smirks and shrugs his shoulders*.

*Spinel: I told you to think twice then three times (before getting the haircut) if needed*.

*He laughs for a long time and points his index finger at his own fringe*.

*Spinel: But then again, it's not all too… yes it is too bad*.

*Ivory covers her face with her hands and makes a crying sound*.

(Mintrack, May 12: Observation notes).

The interaction shows humor that is used to undermine another person or to reinforce their lower status. In his interview Spinel (the manager) further revealed:

*I can only joke well when there is a suitable target. My style of humor is really about picking on others, so when there is a target that can be smashed and go under (me) the situation works out well*. (Spinel, 31, Mintrack).

Spinel reveals his feeling of superiority [“*a target that can be smashed and go under (me)*”], suggesting that he considers his humor successful when it heightens his own social position, at the victim's expense. His role as manager gives him autonomy that allows him to openly denigrate Ivory (“*you look really stupid*”) as she is lower in the hierarchy. His power is unchallenged in his put-down jokes and the other employees mimic his words to reinforce and escalate the joke until Ivory signals defeat by making a “*crying sound*.” Her participation in the joke by pretending to cry, saves face as she is forced to play along and participate in the joke at her own expense but such humiliation and barbed humor surely impacts her PWB although she gamely plays along with the joke as expected.

Humor may also be used to maintain *chemyon* when a person feels ashamed. Observation incidents captured both verbatim conversations and some body language of organizational participants. Humor may mask or hide or ignore embarrassing moments or workplace mistakes. This observation includes three female workers from Mintrack (Citrine, Emerald, and Ivory), spending their lunchtime drawing pictures:

*Ivory spills the contents from her large pencil case, picking up a small, silver color craft knife. She turns around to Citrine, who is busy drawing her own picture, and offers to sharpen Citrine's pencil with the knife. As Citrine passes the pencil to Ivory, Ivory blurts out quite loudly:*

“*I'm good at sharpening pencils, I'm good at sharpening pencils!”*

*Ivory slices away the wooden pieces from the pencil on a paper positioned on her lap, but the sharpened pencil is not “sharp,” but rather bulky. Citrine grabs her pencil from Ivory and starts to sharpen it again herself. Ivory stares at Citrine*.

*Ivory: (Whispers) Oh my gosh, I better practice my pencil sharpening skills to seduce your hearts!*

*Both Citrine and Emerald do not respond to Ivory's comment. Ivory smiles, faces down, and starts to pick on her fingers*. (Mintrack, April 17: Observation notes).

Ivory attempts to cope with her embarrassment/shame by using hyperbolic humor *(“I better practice my pencil sharpening skills to seduce your hearts*”). It appears that Ivory uses humor to reduce the embarrassment she feels in failing to sharpen the pencil. She considers this situation to be shameful, as she fails in accomplishing a task which she has confidently announced to her seniors. Ivory's body language also indicates that she feels shame as she maintains her smile but bows her head and nervously picks her fingers. When questioned afterwards Ivory tersely stated that “*it was embarrassing*” and refused to respond further. It seems that her humorous remark—“*seducing your hearts*” was intended to divert her colleague's attention away from her “shame,” using humor to save face. Her unwillingness to talk about this embarrassment leaves us not knowing whether her face-saving humor was successful in releasing her painful feelings.

Humor is used to save face in these Korean workplace interactions. We see this through participants making a hyperbolic humorous comments to cover embarrassment, and playing along within a barbed joke about a new haircut. Although we achieved some follow-up interviews to these incidents it was confronting for our Korean participants to discuss these interactions as they indicated that they experienced shame that made them uncomfortable. Interviews did elicit some harsh comments from one manager who openly declared his objective of “smashing” and “picking on” his subordinates, deliberately shaming them through targeted “humor.” These hierarchical dynamics of the “shamer” and the “shamed” will be explored relation to PWB in the subsequent discussion.

## Discussion

The constructive effect of humor has been emphasized in many past studies, especially with regards to its positive emotional, relational, and communicational influences [see Alden et al. (1993); Holmes (2000), and Martin (2004)]. Humor is a contextual phenomenon that is perceived differently in different cultural contexts. Humor may be approached differently in Western and Eastern contexts and Confucian-based cultures may not favor humor in social interactions (Yue et al., 2016). Korean society embeds strong Confucian values, and organizations reflect such societal ideas in forms of organizational hierarchy (Rowley and Bae, 2003). Our data corroborates prior studies finding that humor does occur in Korean organizations (Kim and Lee, 2009; Jung, 2014) but we contribute new ideas to the limited research in showing how humor in Korean organizations is constrained by Confucian values of hierarchy and respect with conflicting influences on PWB.

We argue that most humor in these Korean workplaces is instigated by those with superior hierarchical status (managers) determined by role, tenure and age. Our participants showed that humor is enacted according to the relational hierarchy of the communicators, where superiors had more freedom to use humor, while subordinates considered humor to be risky or potentially impolite. Building on earlier research (Smith and Powell, 1988) we show how hierarchical differences between organizational members influences how humor is enacted. We argue that humor is riskier and potentially more damaging for subordinate employees who may consider humor to be inappropriate, as Confucian values teach people to behave seriously, formally (King and Bond, 1985), and show obedience (Lim, 1999). We argue that this may even compel subordinate employees to feign amusement to managers' humor.

Our younger, subordinate participants emphasized the cultural value of politeness and perceived humor as a potentially impolite behavior in their workplace and somewhat risky for them. However, when the humor is top-down and instigated by a manager, then politeness norms influence a subordinate employee to at least appear to appreciate manager's humor and display an expected response such as laughter or a smile- even if they are not amused. In applying Ryff's (1989) dimensions to our data it appears that the dimensions of autonomy and positive relations with others are seminal to the PWB of workers in our studies companies. Autonomy in humor seems to reside firmly with managers and those with superior status can joke freely and even make barbed jokes at the expense of their subordinates. While this may increase PWB for the managers offering them a release through joking and allowing them to alleviate their feelings and stress, it does not appear to have a comparable benefit for the subordinate employees. They lack autonomy in humor exchanges and therefore their responses are governed by the workplace politeness rules that see them laughing at jokes at their own expense, or forcing laughter at jokes that they do not find funny. Therefore the humor here may reduce PWB for those in subordinate positions as their autonomy is highly constrained by politeness norms and therefore both their response to humor and their creation of humor is governed by norms of polite behavior [see Kadar and Spencer-Oatey (2016)].

In one of our observations we saw a senior manager use honorifics in his joke to redefine his relationship with a soon-to-be exiting subordinate. The importance of honorifics used in everyday work conversations (including humor interactions) are linked to linguistic politeness in Japanese and Korean societies (Shibamoto-Smith, 2011; Brown et al., 2014). Zajdman (1995) identified that joking may be used to promote distance. In our example, honorifics were employed as a distancing mechanism rather than a signal of politeness and although framed in humor, the sarcastic honorifics signified that the subordinate was now an “outsider.” Therefore, honorifics may not always serve as polite behavior, but can be face-threatening in targeted humor that redefines workplace relationships. While this may have again, offered the manager a way to release his feelings about the departure of his employee that may have increased his PWB, he may have simultaneously embarrassed and distressed the leaving employee, at the expense of the subordinate's PWB.

Much of the humor we encountered served to save face (chemyon) and we saw humor used to disguise mistakes, offset embarrassment and redirect a joke's target. An early study identified that Japanese people experienced mistakes and predicaments as shame (Imahori and Cupach, 1994) and our respondents indicated that in some of these humor interactions they felt shame. Shame is a self-conscious emotion that can signal when a person has violated moral standards or social norms of their (work) group. Shame can include self-criticism and ruminative thoughts, increasing the need for emotional support and can adversely affect psychological well-being (Kiffin-Petersen, 2018). The concept of *shame* is linked to chemyon, with our participants articulating their shame and embarrassment even for minor mistakes. Humor helped some to try and mitigate and save face after “shameful” mistakes but somewhat disturbingly, a manager openly declared that he used humor to shame and “pick on” his subordinates, potentially causing them distress and embarrassment.

Saving face through humor is strongly linked to hierarchical dynamics and relationships as lower level employees sought to placate senior managers, senior managers sought to take back their superior position, and even used humor to denigrate and belittle subordinates while re-establishing dominance and their superior position. In contrast to Western literature that argues that humor can be used by subordinates to safely challenge managers' directives (Holmes and Marra, 2002; Plester and Orams, 2008), we found that subordinate Korean employees were tightly bound by politeness rules that constrained humor use toward their higher level managers, making humor instigation too risky for most lower level workers. However, the same politeness dynamic dictated a polite response to humor instigated by a senior manager and amusement had to be displayed by subordinates—even if feigned. In Western organizations the liberating freedom and momentary escape enjoyed by jokes at authority seems a release not afforded to all of their Korean counterparts, and some of our examples indicated that our Korean participants were aware of these East-West differences. Therefore the maintenance of positive relationships important to improved PWB guided these workers' humor responses. Relational harmony superseded the notion of autonomy, also significant to PWB (Ryff, 1989), in how humor was enacted in these workplaces.

Korean organizational contexts are highly complex due to the hierarchical relationship structure and the use of honorifics, which convey hidden meanings. We argue that humor interactions help us to understand the important hierarchical relational dynamics that are prioritized in Korean workplaces. Such an understanding identifies some significant implications for workers' PWB as they skillfully navigate complex workplace dynamics and preserve their own and others' psychological well-being.

## Conclusion and Implications

This study explores relationships between humor, hierarchy and psychological well-being in Confucian-based Korean workplace contexts. Our findings have implications for both theory and practice. Most significantly, this study offers an original theorization of the significance and prioritization of harmonious relationships over autonomous responses to humor at work. This has implications for PWB and implies that humor can enhance PWB on one dimension whilst simultaneously diminishing a different dimension of PWB. Our study shows that humor used by senior managers may enhance or damage their subordinate's chemyon (face) and we suggest that this may create some significant implications for subordinate employees' equilibrium and well-being. Additionally, managers might consider their impact on employee well-being and frame their humor to be more supportive toward employees. We find that honorifics are implicated in humor interactions with subordinates and they work to establish, maintain and redefine hierarchical workplace relationships.

In terms of practice, we argue that workers must consider key cultural differences in workplace humor that impact on workplace relationships and individual well-being. The actual form of language used to engage in humor (such as honorifics) may not be the most important aspect to consider, but the cultural expectations, relational hierarchy, and the associated and expected responses should be carefully considered in humor interactions. It seems that reactions to humor interactions may be important in maintaining harmonious work relationships and our participants' career progressions, both important to their on-going PWB.

Although not generalizable, our study provides significant insights into worker perspectives and experiences at different hierarchical levels and across different Korean organizations. We call for a wider research agenda into intricate Eastern organizational experiences and argue that studying humor interactions offers rich and novel insights into employee and managerial relational dynamics that contribute to PWB, on-going cultural understandings and theorizations.

## Data Availability Statement

The dataset presented in this article are not readily available because of privacy and ethical issues. Requests to access the dataset should be directed to hs-kim@yonsei.ac.kr.

## Ethics Statement

The studies involving human participants were reviewed and approved by University of Auckland Human Participants Ethics Committee. The patients/participants provided their written informed consent to participate in this study.

## Author's Note

This article is substantively and empirically distinct from Kim and Plester (2019). Both papers are based on the same data set (and thus the same number of interviews and observations). However, our two papers provide different interpretations and thus different overall findings. Our first paper focuses on the different dimensions of hierarchy within the studied Korean organizations in relation to humor, especially for those in subordinate positions. This current paper focuses on honorifics, politeness, and “face” in humor. Hierarchy is a part of the context in analyzing these ideas, rather than the main outcome, thus we discuss the implications on PWB for parties involved.

## Author Contributions

All authors shared the writing of this paper and contributed evenly to the writing and development of the work.

## Conflict of Interest

The authors declare that the research was conducted in the absence of any commercial or financial relationships that could be construed as a potential conflict of interest.

## Publisher's Note

All claims expressed in this article are solely those of the authors and do not necessarily represent those of their affiliated organizations, or those of the publisher, the editors and the reviewers. Any product that may be evaluated in this article, or claim that may be made by its manufacturer, is not guaranteed or endorsed by the publisher.

## References

[B1] Alden D. L. Hoyer W. D. Lee C. (1993). Identifying global and culture-specific dimensions of humor in advertising: a multinational analysis. J. Market. 57, 64–75. 10.1177/002224299305700205

[B2] Alvesson M. (2010). Interpreting Interviews. London: Sage. 10.4135/9781446268353

[B3] Alvesson M. Sköldberg K. (2000). Reflexive Methodology. New Vistas for Qualitative Research. London: Sage

[B4] Auerbach C. Silverstein L. B. (2003). Qualitative Data: An Introduction to Coding and Analysis. New York, NY: NYU Press.

[B5] Azevedo G. (2020). Does organizational nonsense make sense? laughing and learning from French corporate cultures. J. Manag. Inquiry 29, 385–403. 10.1177/1056492618813203

[B6] Berger A. A. (1987). Humor an introduction. Am. Behav. Sci. 30, 6–15. 10.1177/000276487030003002

[B7] Billig M. (2005). Laughter and Ridicule: Towards a Social Critique of Humour. Thousand Oaks, CA: Sage.

[B8] Bitterly T. B. Brooks A. W. Schweitzer M. E. (2017). Risky business: when humor increases and decreases status. J. Pers. Soc. Psychol. 112:431. 10.1037/pspi0000079 27831701

[B9] Braun V. Clarke V. (2006). Using thematic analysis in psychology. Qualitat. Res. Psychol. 3, 77–101. 10.1191/1478088706qp063oa

[B10] Brown L. (2011). “Korean honorifics and ‘revealed', ‘ignored' and ‘suppressed' aspects of Korean culture and politeness,” in Politeness Across Cultures, eds F. Bargiela-Chiappini and D. Kadar (London: Palgrave Macmillan), 106–127. 10.1057/9780230305939_6

[B11] Brown L. Winter B. Idemaru K. Grawunder S. (2014). Phonetics and politeness: perceiving Korean honorific and non-honorific speech through phonetic cues. J. Pragmat. 66, 45–60. 10.1016/j.pragma.2014.02.011

[B12] Brown P. Levinson S. C. (1978). “Universals in language usage: politeness phenomena,” in Questions and Politeness: Strategies in Social Interaction, ed E. N. Goody (Cambridge: Cambridge University Press), 56–311.

[B13] Bryman A. Bell E. (2015). Business Research Methods. Oxford: Oxford University Press.

[B14] Butler N. (2015). Joking aside: theorizing laughter in organizations. Cult. Org. 21, 42–58. 10.1080/14759551.2013.799163

[B15] Cann A. Collette C. (2014). Sense of humor, stable affect, and psychological well-being. Eur. J. Psychol. 10, 464–479. 10.5964/ejop.v10i3.746

[B16] Cheung C. K. Yue X. D. (2012). Sojourn students' humor styles as buffers to achieve resilience. Int. J. Intercult. Relat. 36, 353–364. 10.1016/j.ijintrel.2011.10.001

[B17] Chiang H. H. Chien L. H. Lin J. S. Yeh Y. H. Lee T. S. H. (2013). Modeling psychological well-being and family relationships among retired older people in Taiwan. Int. J. Mental Health Nurs. 22, 93–101. 10.1111/j.1447-0349.2012.00840.x 22957966

[B18] Choi Y. (2010). “The history of confucianism in Korea,” in Confucianism in Context: Classic Philosophy and Contemporary Issues, East Asia and Beyond, ed W. Chang (Albany, NY: State University of New York Press), 33–52.

[B19] Cooper C. (2008). Elucidating the bonds of workplace humor: a relational process model. Hum. Relat. 61, 1087–1115. 10.1177/0018726708094861

[B20] Crawford S. A. Caltabiano N. J. (2011). Promoting emotional well-being through the use of humor. J. Posit. Psychol. 6, 237–252. 10.1080/17439760.2011.577087

[B21] Creswell J. W. (2003). Research Design. Qualitative, Quantitative and Mixed Methods Approaches, 2nd Edn. Thousand Oaks, CA: Sage.

[B22] Cunliffe A. Helin J. Luhman J. T. (2014). “Mikhail Bakhtin,” in The Oxford Handbook of Process Philosophy and Organization Studies, eds J. Helin, T. Hernes, D. Hjorth, and R. Holt (Oxford: OUP Oxford),333–347.

[B23] Davis J. M. (2016). Satire and its constraints: case studies from Australia, Japan, and the People's Republic of China. Int. J. Humor Res. 29, 197–221. 10.1515/humor-2015-0080

[B24] Deardorff D. K. (2015). Intercultural competence: mapping the future research agenda [Editorial]. Int. J. Intercult. Relat. 48, 3–5. 10.1016/j.ijintrel.2015.03.002

[B25] Denzin N. Lincoln Y. (2005). The Sage Handbook of Qualitative Research, 3rd edn. Sage: Thousand Oaks.

[B26] der Kinderen S. Valk A. Khapova S. N. Tims M. (2020). Facilitating eudaimonic well-being in mental health care organizations: the role of servant leadership and workplace civility climate. Int. J. Environ. Res. Public Health 17:1173. 10.3390/ijerph17041173 32059592 PMC7068487

[B27] Deuchler M. (1992). The Confucian Transformation of Korea: A Study of Society and Ideology. Cambridge: Harvard Univ. Asia Center.

[B28] Duncan J. B. (2002). “Uses of confucianism in modern Korea,” in Rethinking Confucianism. Past and Present in China, Japan, Korea, and Vietnam, eds B. A. Elman, J. B. Duncan, and H. Ooms (Los Angeles, CA: UCLA Asian Pacific Monograph Series), 431–462.

[B29] Evans J. B. Slaughter J. E. Ellis A. P. Rivin J. M. (2019). Gender and the evaluation of humor at work. J. Appl. Psychol. 104:1077. 10.1037/apl0000395 30730166

[B30] Freud S. (1960). Jokes and Their Relation to the Unconscious. Translated by J. Strachey. Routledge and Kegan Paul PLC.

[B31] Gale J. Vance C. M. (2012). Cross-cultural knowledge sharing for competitive advantage: an interview with Nigel J. Holden. J. Manage. Inq. 21, 397–403. 10.1177/1056492612441394

[B32] Hatch M. J. (2012). Bringing culture back from institutional Siberia. J. Manag. Inq. 21, 84–87. 10.1177/1056492611419791

[B33] Hofstede G. (1984). Culture's Consequences: International Differences in Work-Related Values. Newbury Park, CA: Sage.

[B34] Holmes J. (2000). Politeness, power, and provocation: how humor functions in the workplace. Discour. Stud. 2, 159–185. 10.1177/1461445600002002002

[B35] Holmes J. (2006). Sharing a laugh: pragmatic aspects of humor and gender in the workplace. J. Pragmat. Spec. Issue Gender Humor 38, 26–50. 10.1016/j.pragma.2005.06.007

[B36] Holmes J. Marra M. (2002). Having a laugh at work: how humor contributes to workplace culture. J. Pragmat. 34, 1683–1710. 10.1016/S0378-2166(02)00032-2

[B37] House J. Kasper G. (1981). “Politeness markers in English and German,” in Conversational Routine, ed F. Coulmas (Mouton: The Hague), 157. 10.1515/9783110809145.157

[B38] Hwang S. J. J. (1991). Terms of address in Korean and American cultures. Intercult. Commun. Stud. 1, 117–136.

[B39] Imahori T. T. Cupach W. R. (1994). A cross-cultural comparison of the interpretation and management of face: US American and Japanese responses to embarrassing predicaments. Int. J. Intercult. Relat. 18, 193–219. 10.1016/0147-1767(94)90028-0

[B40] Isen A. M. Daubman K. A. Nowicki G. P. (1987). Positive affect facilitates creative problem solving. J. Personal. Soc. Psychol. 52, 1122–1131. 10.1037/0022-3514.52.6.1122 3598858

[B41] Jiang T. Li H. Hou Y. (2019). Cultural differences in humor perception, usage, and implications. Front. Psychol. 10:123. 10.3389/fpsyg.2019.00123 30761053 PMC6361813

[B42] Johnson S. Robertson I. Cooper C. L. (eds.). (2018). “Work and well-being,” in Well-Being (Cham: Palgrave Macmillan), 89–108. 10.1007/978-3-319-62548-5_7

[B43] Jung S. H. (2014). Impact of supervisor's humor on civil servant's innovative work behavior. J. Korea Contents Assoc. 14, 733–743. 10.5392/JKCA.2014.14.12.733

[B44] Kadar D. Z. Spencer-Oatey H. (2016). The bases of (im)politeness evaluations: culture, the moral order and the East-West debate. East Asian Pragmat. 1, 73–106. 10.1558/eap.v1i1.29084

[B45] Kiffin-Petersen S. A. (2018). Ashamed of Your Shame? How discrepancy self-talk and social discourse influence individual shame at work. In Social Functions of Emotion and Talking About Emotion at Work. Edward Elgar Publishing. 10.4337/9781786434883.00018

[B46] Kim H. S. Plester B. A. (2019). Harmony and distress: humor, culture, and psychological well-being in South Korean organizations. Front. Psychol. 9:2643. 10.3389/fpsyg.2018.02643 30666223 PMC6330304

[B47] Kim K. H. (2018). Enhancing solidarity through dispreferred format: the nuntey-clause in Korean conversation as a normative basis for leveraging action. East-Asian Pragmat. 3, 27–57. 10.1558/eap.34742

[B48] Kim T. Y. Lee D. R. (2009). A Sense of Humor and Performance at Work: The Mediating Effects of Self-Efficacy. SSRN Scholarly Paper ID 2638762. Rochester, NY: Social Science Research Network. Available online at: https://papers.ssrn.com/abstract=2638762 (accessed May 05, 2021).

[B49] King A. Y. Bond M. H. (1985). “The Confucian paradigm of man: a sociological view,” in Chinese Culture and Mental Health, eds W. S. Tseng and D. Y. Wu (Orlando, FL: Academic Press), 29–46. 10.1016/B978-0-12-701630-6.50009-5

[B50] Kuiper N. A. Grimshaw M. Leite C. Kirsh G. (2004). Humor is not always the best medicine: specific components of sense of humor and psychological well-being. Humor 17, 135–168. 10.1515/humr.2004.002

[B51] La Fave L. Haddad J. Maesen W. A. (1976). “Superiority, enhanced self-esteem and perceived incongruity humor theory,” in Humor and Laughter: Theory, Research, and Applications, eds A. J. Chapman and H.C. Foot (London: John Wiley and Sons), 63–92. 10.4324/9780203789469-5

[B52] Lefcourt H. M. Martin R. A. (1986). Humor and Life Stress: Antidote to Adversity. New York, NY: Springer-Verlag. 10.1007/978-1-4612-4900-9

[B53] Lim T. (1999). Hankukin-Ui Communication Gach'ikwan: Chont'ong-Gwa Byonhwa communication beliefs and values in Korea: tradition and change. J. Commun. Assoc. Korea 7, 52–66.

[B54] Lun V. M. C. Bond M. H. (2006). Achieving relationship harmony in groups and its consequence for group performance. Asian J. Soc. Psychol. 9, 195–202. 10.1111/j.1467-839X.2006.00197.x

[B55] Marra M. Holmes J. (2007). Humour across cultures: joking in the multicultural workplace. Handb. Intercult. Commun. 153:172. 10.1515/9783110198584.2.153

[B56] Martin D. M. (2004). Humor in middle management: women negotiating the paradoxes of organizational life. J. Appl. Commun. Res. 32, 147–170. 10.1080/0090988042000210034

[B57] Martin R. Ford T. E. (2018). The Psychology of Humor. An Integrative Approach, 2nd Edn. London: Academic press.

[B58] Martin R. A. (2006). The Psychology of Humor. Burlington, MA: Elsevier.

[B59] Martin R. A. Kuiper N. A. Olinger L. J. Dance K. A. (1993). Humor, coping with stress, self-concept, and psychological well-being. Humor 6:89. 10.1515/humr.1993.6.1.89

[B60] Martin R. A. Puhlik-Doris P. Larsen G. Gray J. Weir K. (2003). Individual differences in uses of humor and their relation to psychological well-being: development of the Humor Styles Questionnaire. J. Res. Personal. 37, 48–75. 10.1016/S0092-6566(02)00534-2

[B61] McBrian C. D. (1978). Language and social stratification: the case of a confucian society. Anthropol. Linguist. 20, 320–326.

[B62] McCreaddie M. Payne S. (2014). Humour in health-care interactions: a risk worth taking. Health Expect. 17, 332–344. 10.1111/j.1369-7625.2011.00758.x 22212380 PMC5060733

[B63] McSweeney B. (2002). Hofstede's model of national cultural differences and their consequences: a triumph of faith - a failure of analysis. Hum. Relat. 55, 89–118. 10.1177/0018726702551004

[B64] Morreall J. (1983). Taking Laughter Seriously. SUNY Press.

[B65] Park J. S. (1993). “Yeh” communication: confucian thoughts and human communication in Korea. J. Kor. Media Commun. 30, 59–97.

[B66] Plester B. (2009). Healthy humour: using humour to cope at work. Kōtuitui 4, 89–102. 10.1080/1177083X.2009.9522446

[B67] Plester B. A. (2016). The Complexity of Workplace Humor. Laughter, Jokers, and the Dark Side of Humor. London: Springer. 10.1007/978-3-319-24669-7

[B68] Plester B. A. Orams M. (2008). Send in the clowns: the role of the joker in three New Zealand IT companies. Humor Int. J. Humor Res. 21, 253–281. 10.1515/HUMOR.2008.013

[B69] Plester B. A. Sayers J. (2007). “Taking the piss”: functions of banter in the IT industry: humor. Int. J. Humor Res. 20, 157–187. 10.1515/HUMOR.2007.008

[B70] Ridanpää J. (2019). Crisis events and the inter-scalar politics of humor. GeoJournal 84, 901–915. 10.1007/s10708-018-9900-5

[B71] Robertson I. Cooper C. (2011). Well-being: Productivity and Happiness at Work. Basingstoke: Palgrave Macmillan. 10.1057/9780230306738

[B72] Rowley C. Bae J. (2003). “Culture and management in South Korea,” in Culture and Management in Asia, ed M. Warner (London: Routledge Curzon), 187–209.

[B73] Ruch W. (2008). “Psychology of humor,” in The Primer of Humor Research, ed V. Raskin (New York, NY: Mouton de Gruyter), 17–100. 10.1515/9783110198492.17

[B74] Ruch W. Wagner L. Heintz S. (2018). Humor, the PEN model of personality, and subjective well-being: support for differential relationships with eight comic styles. Riv. Ital. Stud. Umorismo 1, 31–44.

[B75] Ruch W. L. Heintz S. (2018). Humor, the PEN model of personality, and subjective well-being: support for differential relationships with eight comic styles. Rivista Italiana di Studi sull'Umorismo 1, 31–44. Available online at: http://hdl.handle.net/10026.1/15550

[B76] Ryff C. D. (1989). Happiness is everything, or is it? explorations on the meaning of psychological well-being. J. Personal. Soc. Psychol. 57, 1069–1081. 10.1037/0022-3514.57.6.1069

[B77] Ryff C. D. Keyes C. L. M. (1995). The structure of psychological well-being revisited. J. Pers. Soc. Psychol. 69:719. 10.1037/0022-3514.69.4.719 7473027

[B78] Schein E. H. (1985). Organizational Culture and Leadership. San Francisco, CA: Jossey-Bass Publishers.

[B79] Schein E. H. (2004). Organizational Culture and Leadership. San Francisco: Jossey Bass.

[B80] Sharpe T. L. Koperwas J. (2003). Behavior and Sequential Analyses: Principles and Practice. Thousand Oaks, CA: Sage. 10.4135/9781412983518

[B81] Shibamoto-Smith J. S. (2011). Honorifics, “politeness,” and power in Japanese political debate. J. Pragmat. 43, 3707–3719. 10.1016/j.pragma.2011.09.003

[B82] Shim T. Y. Kim M. S. Martin J. N. (2008). Changing Korea: Understanding Culture and Communication. New York, NY: Peter Lang.

[B83] Smith C. M. Powell L. (1988). The use of disparaging humor by group leaders. Southern Speech Commun. J. 53, 279–292. 10.1080/10417948809372729

[B84] Sosik J. J. Chun J. U. Koul R. (2017). Relationships between psychological wellbeing of Thai college students, goal orientations, and gender. Psychol. Schools 54, 703–717. 10.1002/pits.22024

[B85] Stake R. E. (2013). Multiple Case Study Analysis. New York, NY: The Guilford press.

[B86] Tanaka H. (2018). Solo or shared laughter in coparticipant criticism in Japanese conversation. East Asian Pragmat. 3, 125–149. 10.1558/eap.35801

[B87] Upadhyay S. R. (2003). Nepali requestive acts: linguistic indirectness and politeness re-considered. J. Pragmat. 35, 1651–1677. 10.1016/S0378-2166(03)00076-6

[B88] Welch C. Piekkari R. (2006). Crossing language boundaries: qualitative interviewing in international business. Manag. Int. Rev. 46, 417–437. 10.1007/s11575-006-0099-1

[B89] Wijewardena N. Härtel C. E. Samaratunge R. (2017). Using humor and boosting emotions: an affect-based study of managerial humor, employees' emotions and psychological capital. Hum. Relat. 70, 1316–1341. 10.1177/0018726717691809

[B90] Wood R. E. Beckmann N. Rossiter J. R. (2011). Management humor: asset or liability?. Org. Psychol. Rev. 1, 316–338. 10.1177/2041386611418393

[B91] Yamagishi T. Jin N. Miller A. S. (1998). In-group bias and culture of collectivism. Asian J. Soc. Psychol. 1, 315–328. 10.1111/1467-839X.00020

[B92] Yao X. (2000). An Introduction to Confucianism. Cambridge: Cambridge University Press. 10.1017/CBO9780511800887

[B93] Yoon K. J. (2004). Not just words: Korean social models and the use of honorifics. Intercult. Pragmat. 1, 189–210. 10.1515/iprg.2004.1.2.189

[B94] Yue X. Jiang F. Lu S. Hiranandani N. (2016). To be or not to be humorous? cross cultural perspectives on humor. Front. Psychol. 7:1495. 10.3389/fpsyg.2016.01495 27757091 PMC5048456

[B95] Zajdman A. (1995). Humorous face-threatening acts: humor as strategy. J. Pragmat. 23, 325–339. 10.1016/0378-2166(94)00038-G

[B96] Zillmann D. (1983). “Disparagement humor,” in Handbook of Humor Research, eds P. McGhee and H. Goldstein (New York, NY: Springer), 85–107.

